# Preponderance of vaccine-preventable diseases hotspots in northern Ghana: a spatial and space-time clustering analysis from 2010 to 2014

**DOI:** 10.1186/s12889-022-14307-1

**Published:** 2022-10-12

**Authors:** Daniel Amoako-Sakyi, Dorcas Obiri-Yeboah, Anthony Ofosu, Kwadwo Asamoah Kusi, Kingsley Osei, Richard Adade, Ebenezer Aniakwaa-Bonsu, Reginald Quansah, John Arko-Mensah, Brodrick Yeboah Amoah, Godwin Kwakye-Nuako, Eric Yaw Frimpong, Mariama Combasseré-Cherif, Hidaya Mohammed, Boubacar Maiga, Julius Fobil, Isabella Quakyi, Ben A. Gyan

**Affiliations:** 1 Department of Microbiology and Immunology, School of Medical Sciences, College of Health and Allied Sciences, University of Cape Coast, Cape Coast, Ghana; 2 Centre for Health Information Management, Ghana Health Services, Accra, Ghana; 3 Immunology Department, College of Health Sciences, Noguchi Memorial Institute for Medical Research, University of Ghana, Accra, Ghana; 4 Department of Geography and Regional Planning, Faculty of Social Sciences, College of Humanities in Legal Studies, University of Cape Coast, Cape Coast, Ghana; 5 Department of Biological, Environmental and Occupational Health, School of Public Health, College of Health Sciences, University of Ghana, Legon, Ghana; 6 Department of Medical Laboratory Sciences, School of Biomedical and Allied Health Sciences, College of Health Sciences, University of Ghana, Legon, Accra Ghana; 7 Department of Biomedical Sciences, School of Allied Health Sciences, College of Health and Allied Sciences, University of Cape Coast., Cape Coast, Ghana; 8 Centre for Coastal Managenment, University of Cape Coast., Cape Coast, Ghana; 9 Unité de Formation et de Recherche en Sciences et Techniques, Université Nazi, Bobo- Dioulasso, Burkina Faso, Burkina Faso; 10 University of Sciences, Techniques and Technology of Bamako (USTT-B), Bamako, Mali; 11 Office of Population Health and Evaluation, New York State Office of Mental Health, Albany, NY USA

**Keywords:** Vaccine-preventable Diseases, VPD, Ecological zones, Hotspots, Ghana, Artisanal and Small-Scale Gold Mining, ASGM, Immunization

## Abstract

**Background:**

Vaccine-preventable diseases (VPDs) persist globally with a disproportionately high burden in Low and Middle-Income Countries (LMICs). Although this might be partly due to the failure to sustain vaccination coverage above 90% in some WHO regions, a more nuanced understanding of VPD transmission beyond vaccination coverage may unveil other important factors in VPD transmission and control. This study identified VPDs hotspots and explored their relationships with ecology, urbanicity and land-use variations (Artisanal and Small-scale Gold Mining (ASGM) activities) in Ghana.

**Methods:**

District-level disease count data from 2010 to 2014 from the Ghana Health Service (GHS) and population data from the Ghana Population and Housing Census (PHC) were used to determine clustering patterns of six VPDs (Measles, Meningitis, Mumps, Otitis media, Pneumonia and Tetanus). Spatial and space-time cluster analyses were implemented in SaTScan using the discrete Poisson model. *P*-values were estimated using a combination of sequential Monte Carlo, standard Monte Carlo, and Gumbel approximations.

**Results:**

The study found a preponderance for VPD hotspots in the northern parts of Ghana and northernmost ecological zones (Sudan Savannah and Guinea Savannah). Incidence of meningitis was higher in the Sudan Savannah ecological zone relative to: Tropical Rain Forest (p = 0.001); Semi Deciduous Forest (p < 0.0001); Transitional Zone (p < 0.0001); Coastal Savannah (p < 0.0001) and Guinea Savannah (p = 0.033). Except for mumps, which recorded a higher incidence in urban districts (*p* = 0.045), incidence of the other five VPDs did not differ across the urban-rural divide. Whereas spatial analysis suggested that some VPD hotspots (tetanus and otitis media) occur more frequently in mining districts in the southern part of the country, a Mann-Whitney U test revealed a higher incidence of meningitis in non-mining districts (*p =* 0.019). Pneumonia and meningitis recorded the highest (722.8 per 100,000) and least (0.8 per 100,000) incidence rates respectively during the study period.

**Conclusion:**

This study shows a preponderance of VPD hotspots in the northern parts of Ghana and in semi-arid ecoclimates. The relationship between ASGM activities and VPD transmission in Ghana remains blurred and requires further studies with better spatial resolution to clarify.

## Background

Although Global Health Initiatives (GHIs) like the Global Alliance for Vaccines and Immunization (GAVI) and the Expanded Program on Immunization (EPI) have helped reduce the burden of vaccine-preventable diseases (VPDs) in the past decades, worrying evidence of stalling in VPD decline are beginning to emerge [1]. Recent surveillance data suggests VPDs still constitute a significant global health problem, especially in low and middle-income countries (LMICs) [2, 3]. In 2009, it was estimated that diseases that could have been prevented with routine vaccination still caused 2—3 million deaths annually[4]. More than a decade on, VPDs persist with a complicated global outlook even as high-income countries (HICs) record increases in the frequency of outbreaks—a situation that hints at a growing VPD vulnerable population[5].

VPD outbreaks are often attributed to vaccination coverage gaps and the factors underpinning the emergence of these gaps may differ in different regions of the world. In HICs, for instance, a growing anti-vaccine activism is believed to have led to increased nonmedical exemptions from vaccinations, decreased vaccine uptake and increase in VPD incidence[5–8]. Although disinformation surrounding current COVID-19 vaccines has festered some anti-vaccine sentiments in LMICs, gaps in vaccination coverage are mainly due to logistical and financial constraints [9, 10]. It is tempting to pin the persistence of VPDs solely on vaccination coverage gaps but reports of VPD outbreaks in areas with high vaccination coverage [11–13] suggests that other factors such as ecological, climatic, and perhaps land-use changes may directly or indirectly influence VPD transmission[14–18]. Unfortunately, the lack of a nuanced understanding of the interplay between VPD transmission dynamics, ecology, climate, and land-use variations gravely undermines efforts to develop new strategies for targeted interventions [19].

The convergence model of infectious disease transmission elegantly depicts interactions between climate, ecology, and infectious disease transmission patterns[20], but that notwithstanding, very few studies in Ghana have looked at infectious disease transmission through eco-regional lenses[16]. By definition, ecological zones are areas with unified climate, geology, topography, soil, vegetation, and predominant land use[21]. Thus, ecological zones may better explain infectious disease transmission patterns than political or administrative districts. Ghana is home to six distinct terrestrial ecological zones comprising Sudan Savannah, Guinea Savannah, Forest Savannah Transition, Semi-Decideous Rainforest, Rainforest and Coastal Savannah. Plausibly, a better appreciation of the spatial distribution of VPDs among these ecological zones may offer insights into VPD transmission patterns.

Beyond factors in the convergence model of infectious disease transmission, several other factors may influence VPD transmission in unassuming ways. For instance, malnutrition and some occupational-related issues may undermine herd immunity by reducing vaccine-induced immunity against various VPDs even when vaccine coverage is adequate[22]. This brings the relationships between land-use changes and infectious disease transmission into sharp focus. Apart from interfering with complex ecological relationships, human-induced land-use changes such as those caused by the Artisanal and Small-scale Gold Mining (ASGM) industry in some communities in Ghana can indirectly reduce herd immunity for VPDs[20, 23]. Globally, the ASGM sector is thought to be the most significant source of mercury[24] and some communities with ASGM activities have gained notoriety as “toxic sites” because the methods used by miners release immunotoxins such as mercury, arsenic and cadmium into the environment[24]. A study assessing the potential effects of ASGM activities on human health in Ghanaian communities concluded that there was a high certainty that miners and community members have been exposed to high and potentially dangerous levels of mercury, arsenic and cadmium [23]. In the light of an increasing body of literature on immunosuppressive effects of these metals, [25–28] it is logical to hypothesize that inhabitants of such communities with long-term exposure to high levels of these immunotoxins may be immunosuppressed. While the assessment of population-level immunosuppression could prove daunting, comparing VPD clustering patterns in mining and non-mining communities is feasible and may elucidate how mining-related land-use changes affect the transmission dynamics of VPDs in Ghana.

In recent times, epidemiologists have leveraged Geographic Information Systems (GIS) technologies and spatio-temporal modeling techniques to map areas with exceptionally high (hotspots) or low (cold spots) disease rates in different settings. These tools have been used to study diarrhoeal diseases [29–31], several aspects of malaria and tuberculosis epidemiology [32–35], neglected tropical diseases (NTDs) [36] and a host of other conditions of public health importance, including pedestrian crashes [37, 38]. This study used the SaTScan software [39] in mapping six VPDs (measles, meningitis, mumps, otitis media, pneumonia, and tetanus) in Ghana. It also explored the relationships between VPD incidence and ecological zones, settlement type, and gold mining activities. Findings from this study offer vital information on the distribution of VPDs in Ghana and set the stage for further studies that may explain the variable VPD incidence in the country. Managers of the Expanded Programme on Immunization (EPI) and policymakers can draw valuable lessons from the data presented in this study.

## Methods

### Study area

This study was conducted in the West African nation of Ghana, along the Gulf of Guinea, between latitudes 4° and 12°N and longitudes 4°W and 2°E. Ghana occupies a landmass of 238,535 km^2^ and it is bounded to the North by Burkina Faso, the South by the Atlantic Ocean, the East by Togo and to the West by La Cote d’Ivoire. There are currently 16 administrative regions and 275 districts in Ghana. Administrative boundaries in Ghana are often re-demarcated for efficient running. Prior to 2012, Ghana had 10 regions and 170 districts, which were subdivided into 216 districts. These divisions are often done for political expediency and with little recourse to health system and thus, newly created regions and districts often lack complementary disease and demography data. This study was based on the hitherto 10 regions and 170 districts and limited to the period between 2010 and 2014 to ensure data completeness.

### Data sources and preparation

Vaccine-preventable disease count data for a 5-year period (2010–2014) was obtained from the Centre for Health Information Management (CHIM) of the Ghana Health Service (GHS) outfit. The CHIM aggregates disease count data at the district level using data from various health facilities within each district into two main variables- disease count and administrative districts. Population figures for each district were extracted from Ghana’s 2010 PHC data[40]. Both the CHIM and PHC data are useful and reliable data sources that are routinely used in studies of this nature[41]. The population was assumed to be stable during the study period [42]. A shapefile of district boundaries, ecological zones and significant mining areas digitized as polygon features were obtained from the Cartographic Unit of the Department of Geography and Regional Planning, University of Cape Coast.

This study leverages a feature in the PHC dataset that specifies the number of people living in urban and rural areas in a district to determine the settlement type. In classifying districts into settlement types, districts with an urban population greater than the rural population were classified as urban districts, and those with a higher rural population were classified as rural districts. In classifying districts by ecological zones, a map of Ghana with ecological zone boundaries was superimposed on district boundaries. Districts that lay within the boundaries of a particular ecological zone were grouped together and named after that ecological zone. Districts that lay in two or more ecological zones assumed the identity of the ecological zone that occupied the largest landmass of that district. Information from the Ghana Geological Survey Department and the Toxic Sites Identification Program (TSIP) was used in categorizing districts into mining and non-mining. Districts with evidence of mining activities were classified as mining and those without such records of mining activities were classified as non-mining. VPD cluster and incidence maps were generated in ArcMap 10.3 using shapefiles outputs from SaTScan.

### Analytical Approach

This study used the SaTScan software version 9.4.3 to assess high and low rates clusters of VPDs in the study area. Apart from the ease of use, SaTScan has several advantages over similar softwares [39, 43]. SaTScan needs at least three file types to operate: (1) case file containing information on the number of cases per location for a specified period; (2) a population file containing information on the population per location and (3) coordinate file containing information on the coordinates of each location [43]. Grid file may be used if the user is interested in focused cluster testing. Whereas time for each case is required for space-time analysis, SaTScan does not require this for purely spatial analysis and ignores the time even when provided [43]. In this study, purely spatial (henceforth simply referred to as spatial) and space-time cluster analyses were implemented in SaTScan using the Poisson distribution and a maximum spatial cluster size of 50% of the population at risk. Using a maximum cluster size of 50% of the population at risk allows SaTScan to evaluate a wider range of clusters to improve the chances of detecting “true” clusters regardless of their size (i.e. very small or very large). *P*-values for the detected clusters were calculated using the SaTScan default setting. The default settings calculate the *p*-values by using a combination of standard Monte Carlo, sequential Monte Carlo, and Gumbel approximations. We report on the top five clusters in spatial analysis and the top 3 clusters in space-time analysis. To promote a comprehensive and nuanced understanding of the clustering patterns of VPD in the study, we triangulated spatial analysis with appropriate non-spatial statistics (Mann-Whitney U and Kruskal-Wallis H test) and assessed relationships between VPD incidence, settlement types, land-use patterns and ecological zones using IBM SPSS Statistics Version 26. These tests are non-parametric and allow for analysis of non-normally distributed data with minimal cost to statistical power [44].

## Results

### Spatial and space-time clustering of VPDs

**Fig. 1:**
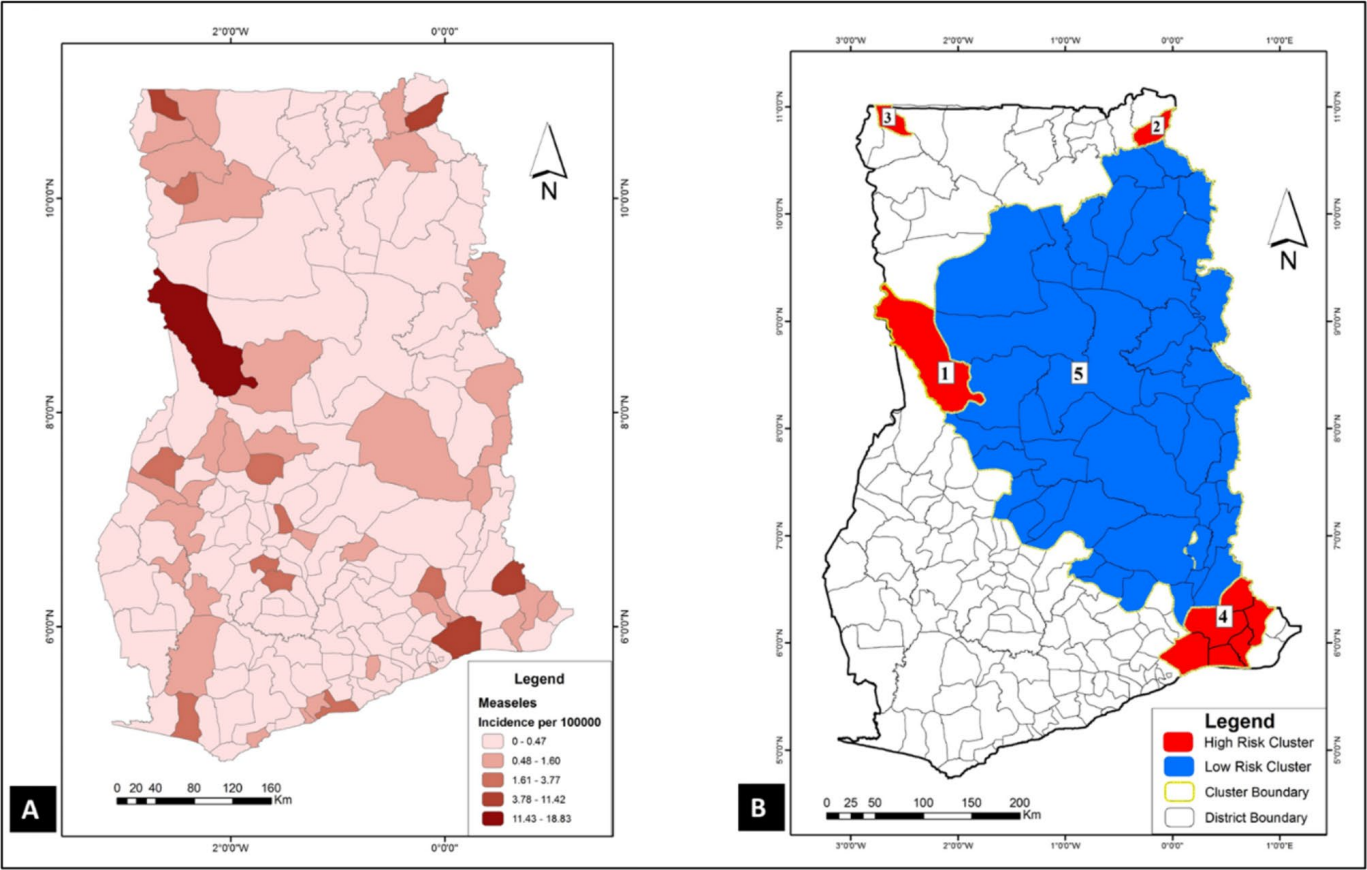
**Choropleths showing the incidence and clustering of measles in the study area** (**A**) A choropleth of measles incidence in the study area (2010–2014); (**B**) Measles cluster map: this choropleth shows the top 5 most likely spatial measles cluster in the study area. Four (4) clusters (1,2,3 and 4) are hotspots (red) whiles a single cluster (5) is a cold spot (blue)

**Fig. 2:**
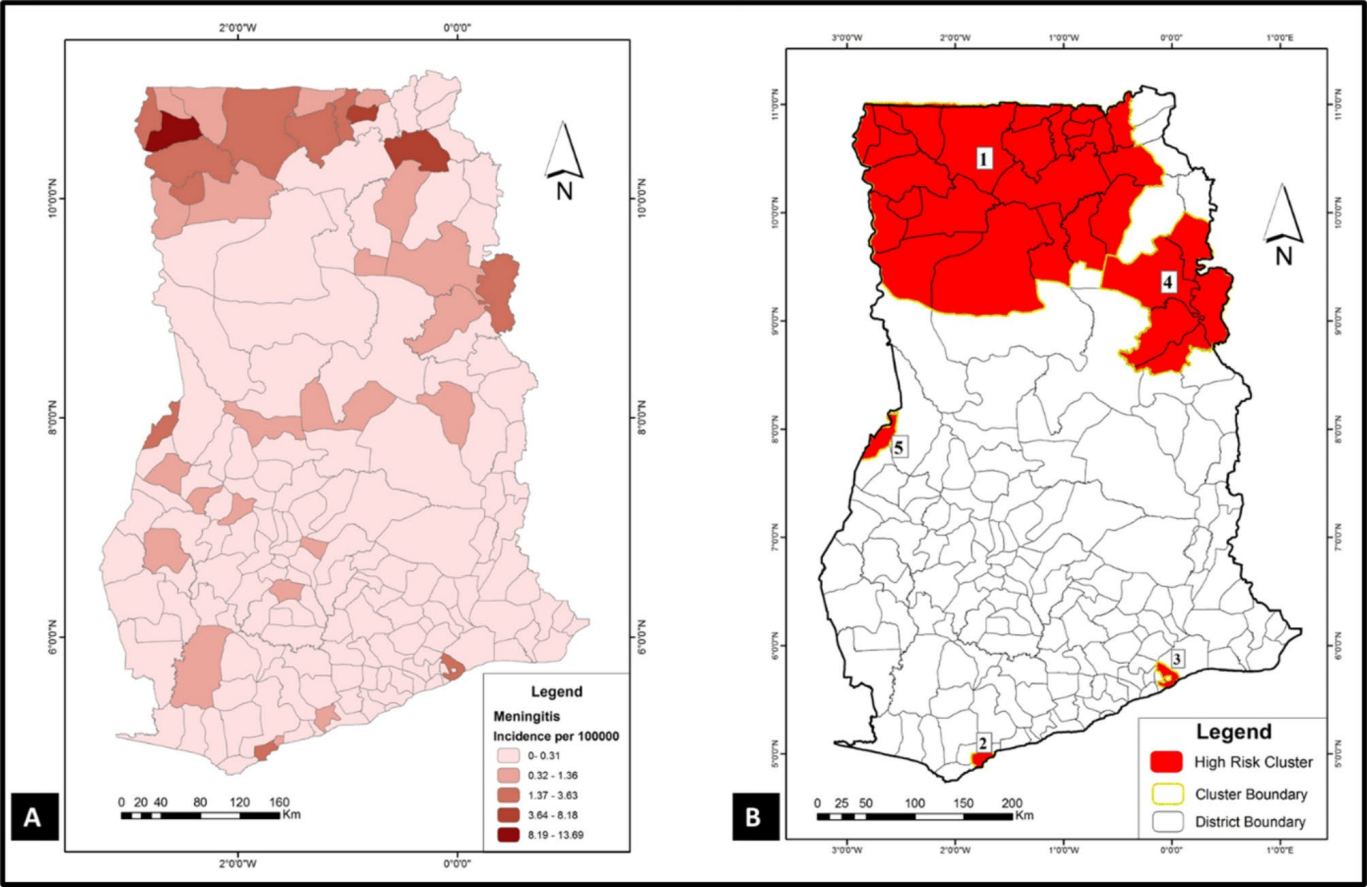
**Choropleths showing the incidence and clustering of meningitis in Ghana** (**A**) A choropleth of meningitis incidence in the study area (2010–2014); (**B**) Meningitis cluster map: this choropleth shows the top 5 most likely spatial meningitis cluster in the study area. All five (5) clusters are hotspots (red)

**Fig. 3:**
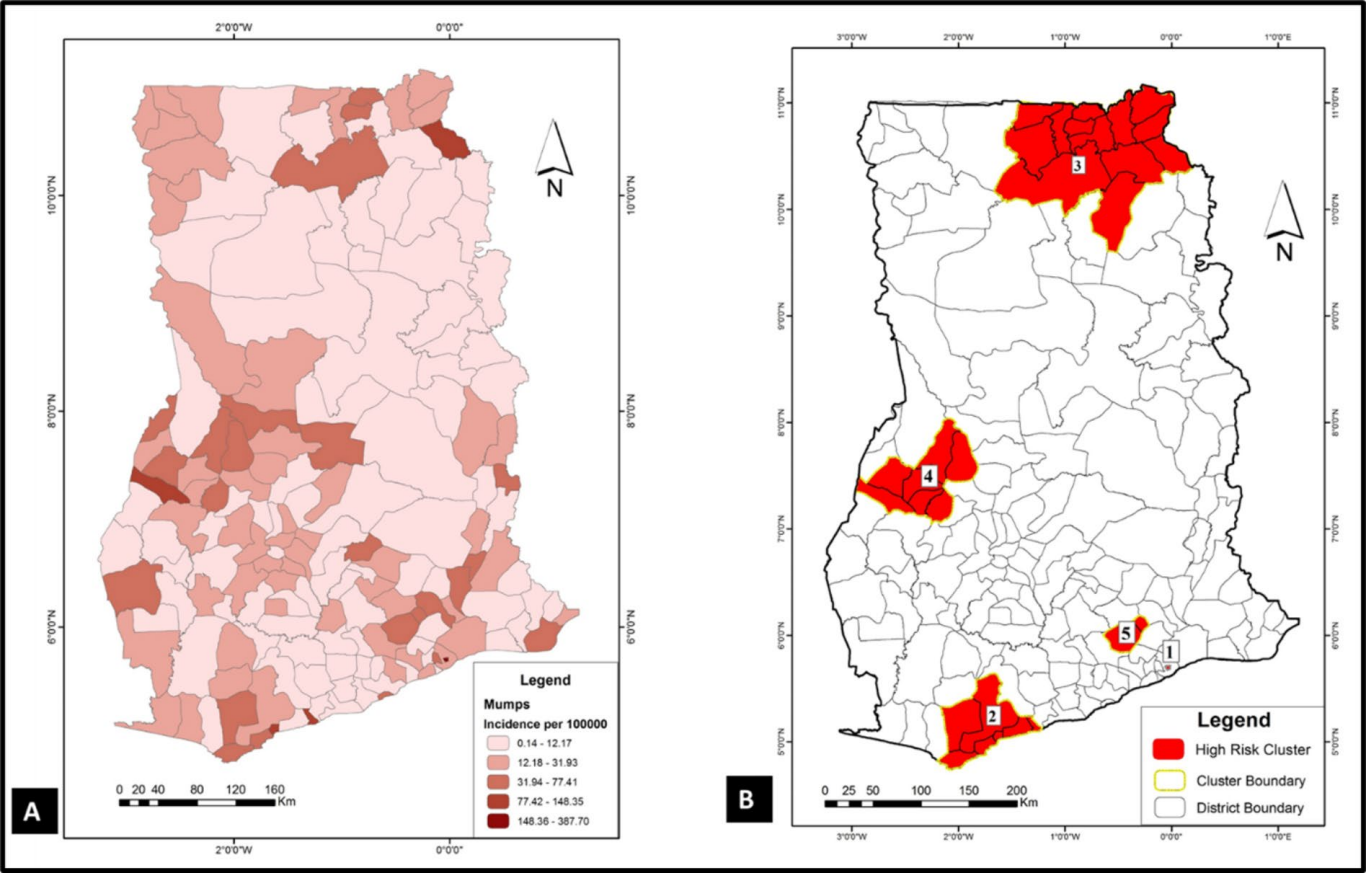
**Choropleths showing the incidence and clustering of measles mumps in Ghana** (**A**) A choropleth of mumps incidence in the study area (2010–2014); (**B**) Mumps cluster map: this choropleth shows the top 5 most likely spatial mumps cluster in the study area. All five (5) clusters are hotspots (red)

**Fig. 4:**
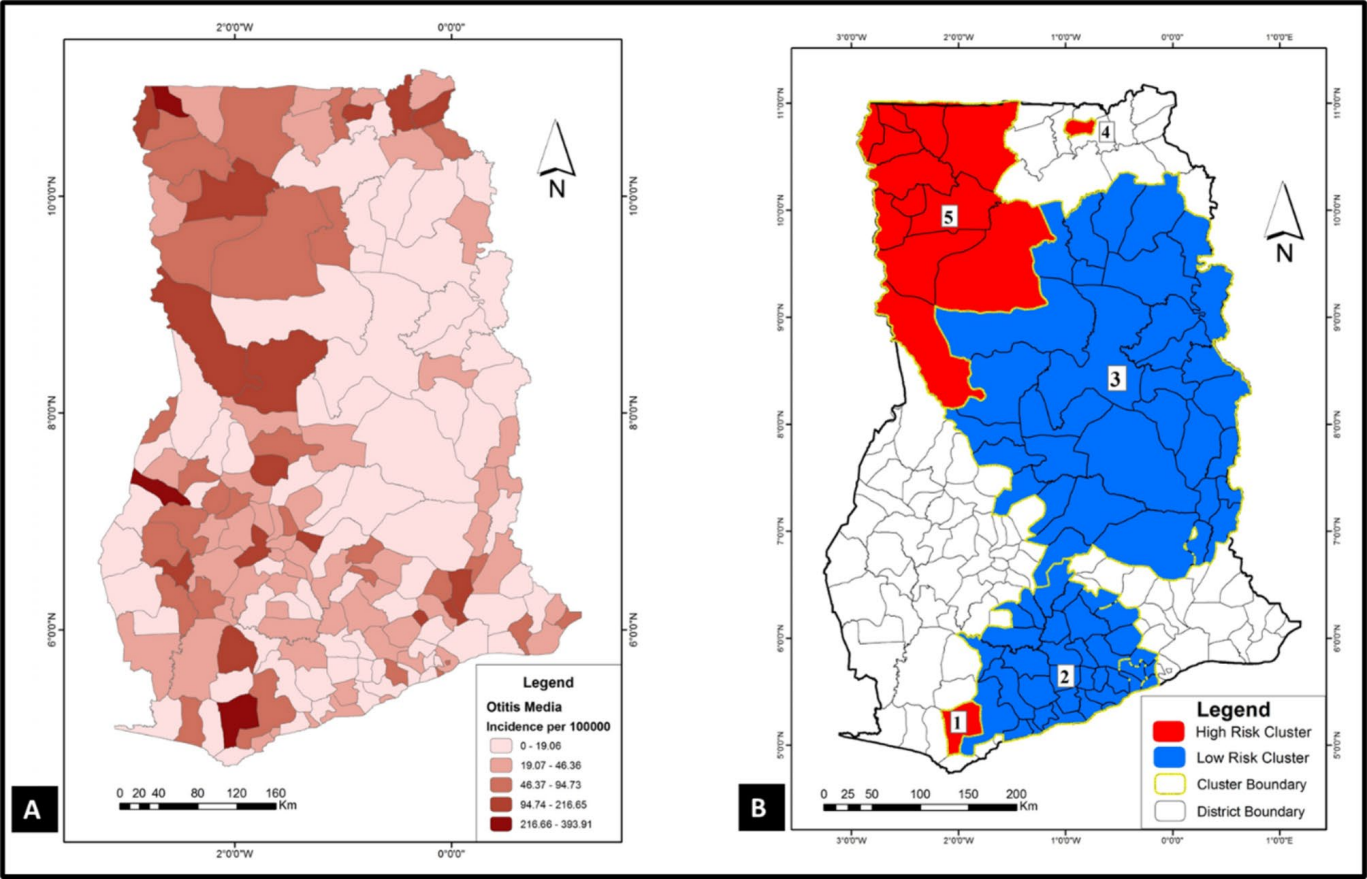
**Choropleths showing the incidence and clustering of otitis media in Ghana** (**A**) Otitis media incidence map for Ghana (2010–2014): this choropleth shows otitis media incidence for each of the 170 districts in Ghana. (**B**) Otitis media cluster map: this choropleth shows the top 5 most likely otitis media clusters in the study area. Three of the clusters (1, 4 and 5) are hotspots (red) whiles two clusters (2 and 3) are cold spots (blue)

**Fig. 5:**
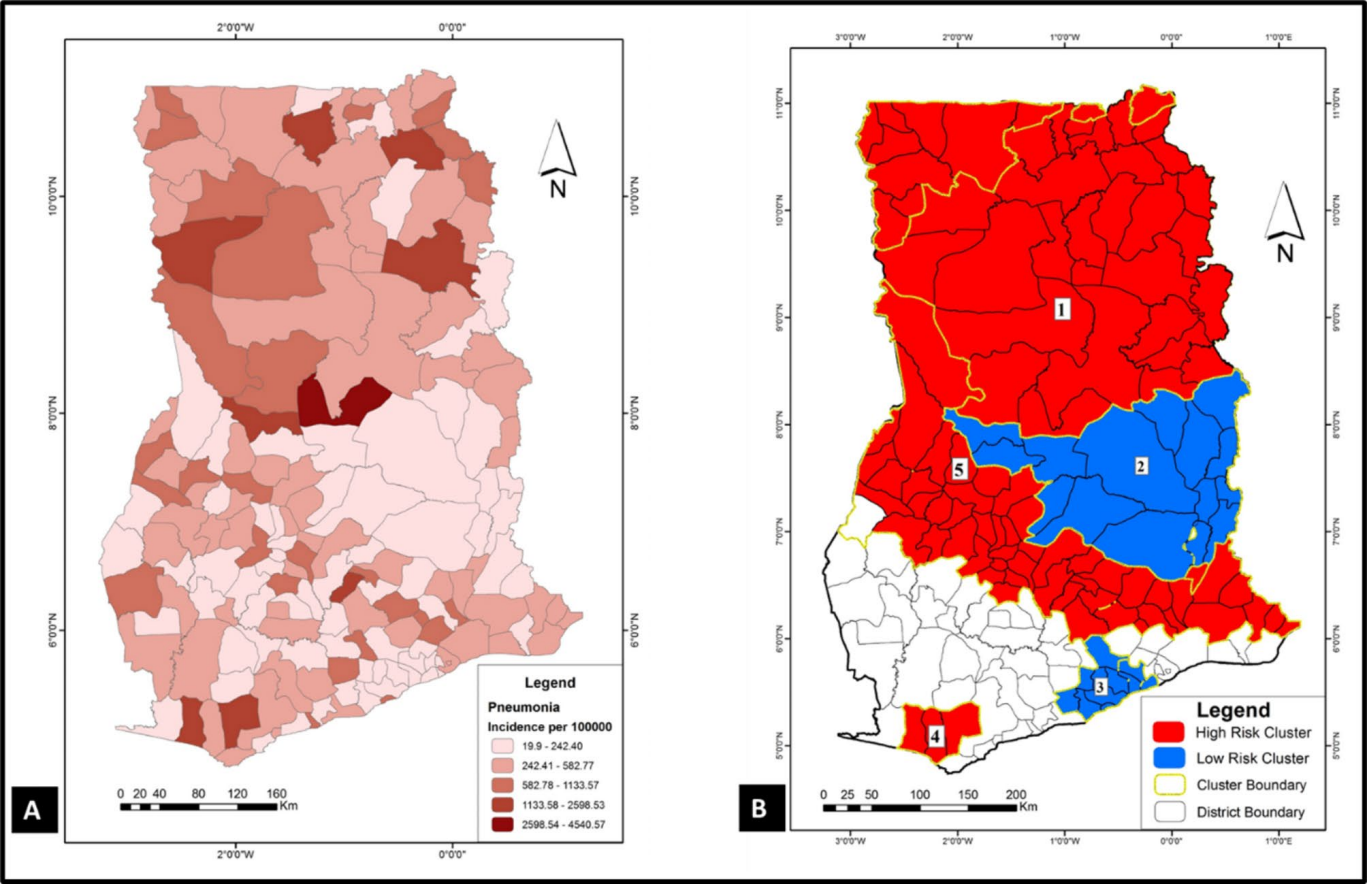
**Choropleths showing the incidence and clustering of pneumonia in Ghana** (**A**) Pneumonia incidence map for Ghana (2010–2014): this choropleth shows pneumonia incidence for each of the 170 districts in Ghana. (**B**) Pneumonia cluster map: this choropleth shows the top 5 most likely pneumonia cluster in Ghana. Three of the clusters (1, 4 and 5) are hotspots (red) whiles two clusters (2 and 3) are cold spots (blue)

**Fig. 6:**
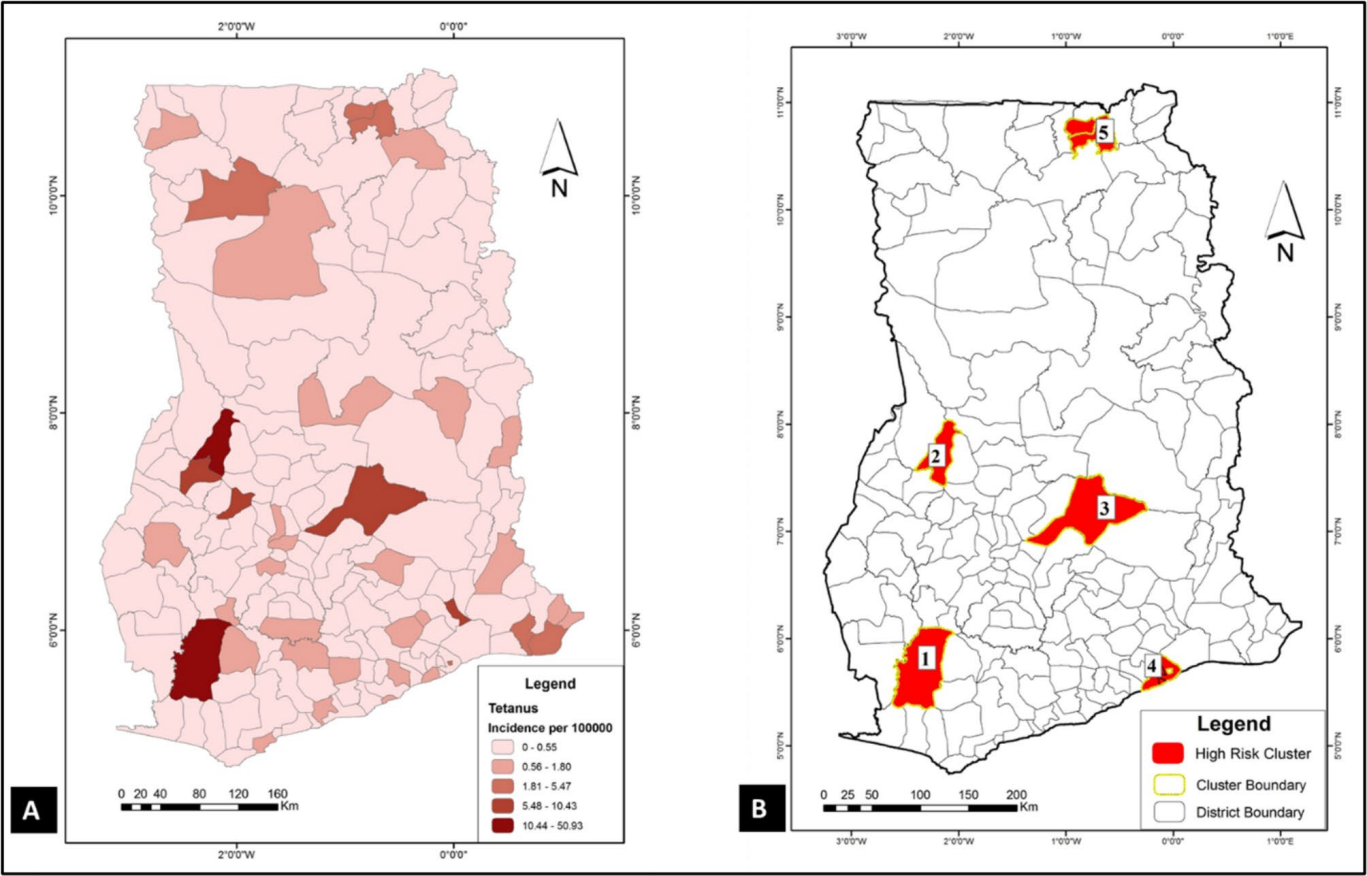
**Choropleths showing the incidence and clustering of tetanus in Ghana** (**A**) Tetanus incidence map for Ghana (2010–2014): Tetanus incidence for each of the 170 districts in Ghana. (**B**) A choropleth showing the top 5 most likely tetanus clusters in Ghana. All 5 cluster had were hotspots (elevated) disease rates

To have a sense of where VPDs clustered in the study area (Ghana), spatial and space-time cluster analyses were implemented in SaTScan using the discrete Poisson model. Results of these analyses were used to generate the choropleths in Figs. 1, 2, 3, 4, 5 and 6. Figure 1 A, 2 A, 3 A, 4 A, 5 and 6 A are incidence maps of VPDs per district for the five-year period (2010–2014). Although incidence maps give some indication of the location of VPD clusters, they are essentially a patchwork of elevated and decreased incidence rates and not particularly revealing of cluster boundaries. Figures 1B, 2B, 3B, 4B, 5B and 6B are cluster maps showing the top 5 most likely clusters for each of the VPDs considered in this study. Cluster attributes, such as cluster type, relative risks, number of cases and expected number of cases, are shown in Table 1. Unless otherwise stated, all reported clusters were significant at a *p* < 0.0001. This study reports the top five most likely spatial clusters and the top three most likely space-time clusters for each VPD.

**Table 1:** Most likely spatial and space-time clusters of the six VPDs.

VPD		Mean Rank		P-value
		**Mining**	**Non-mining**	
**Measles**		77.20	86.30	0.480
**Meningitis**		60.23	87.95	0.019*
**Mumps**		75.87	86.43	0.427
**Otitis media**		103.50	83.76	0.138
**Pneumonia**		95.53	84.55	0.418
**Tetanus**		87.93	85.26	0.836

Top five (5) spatial clusters and top three (3) space-time clusters reported. Spatial and space-time cluster analyses were implemented in SaTScan using Poisson distribution and a maximum spatial cluster size of < 50% of the population at risk. ***P***-values for the detected clusters were calculated using SaTScan’s default setting which estimates *p*-values with a combination of standard Monte Carlo, sequential Monte Carlo, and Gumbel approximations. Administrative districts as the spatial unit of analysis

### Measles:

*Spatial analysis*: A total of 1189 measles cases were recorded during the study period and this corresponds to an incidence rate of 1.0 per 100,000. Top five most likely clusters for measles are shown in Fig. 1B; it comprised four hotspots and a cold spot. The most likely measles cluster was a hotspot that comprised a single district (Bole) in the Northern Region of Ghana (coordinates: 8.717652 N, 2.283088 W). This cluster recorded 116 measles cases and had a relative risk of 43.9 (Table 1). Two of the four other measles hotspots were in the northernmost parts of the country (Fig. 1B) and the only measles hotspot located outside the northern parts of the country was found in the south-eastern parts of Ghana. This measles hotspot was a large cluster (coordinates/radius: (6.177242 N, 0.403307 E) / 41.66 km) comprising five contiguous districts with 111 measles cases and a relative risk of 6.20. The only measles cold spot detected was also the largest and fifth most likely cluster (coordinates/radius: (8.557400 N, 0.299873 E) / 242.22 km). This big cluster comprising 43 districts recorded 98 measles cases and had a relative risk of 0.33.

*Space-time analysis*: To have insights into the temporal dimensions of the detected spatial clusters, a Poisson model space-time analysis was implemented in SaTScan. Information on space-time analysis for all VPDs is shown in Table 1. The top three most likely space-time clusters comprised two hotspots in 2014 and 2012 and a cold spot between 2010 and 2011. Noteworthily, the most likely measles space-time cluster, which occurred in 2014 as a hotspot (relative risk = 214.2) co-located with the most likely spatial cluster (relative risk 43.19) in Bole district. The third space-time cluster was a cold spot identified at (coordinates/radius: (6.216364 N, 0.795332 E) / 267.81 km) between 2010 and 2011 with a relative risk of 0.02.

### Meningitis

*Spatial analysis*: The incidence of meningitis in Ghana for the five-year study period was 0.8 cases per 100,000. The top five most likely meningitis clusters were all hotspots (Fig. 2 A) and the largest of these clusters, which was also the most likely cluster, occurred in the northernmost parts of the country. This cluster (coordinates/radius: (10.656451 N, 1.802392 W) / 159.73 km), comprised 23 districts and had a relative risk of 9.17. The only meningitis clusters located in the southern part of the country (clusters 2 and 3) were single district clusters located in some of the country’s most densely populated districts in the southmost parts of the country (i.e., Tema Metropolitan Assembly and Sekondi Takoradi). The fifth most likely cluster was a single-district cluster located in the Midwest of Ghana and shares boundary with the Ivory coast (coordinates/radius.: (7.916430 N, 2.679788 W) / 0 km).

*Space-time analysis*: The most likely space-time cluster (coordinates/radius: (10.656451 N, 1.802392 W) / 110.90 km) occurred in 2012, comprised 13 districts, and had a relative risk of 33.44. Another space-time cluster (cold spot) (coordinates/radius: (6.908037 N, 2.212044 W) / 221.65 km) emerged between 2010 and 2011, which comprised 103 districts, had zero observed cases instead of the expected 189.89.

### Mumps

*Spatial analysis*: Incidence of mumps in Ghana for the five-year period was 41.2 per 100,000. All five most likely spatial mumps clusters were hotspots and the most likely cluster (coordinates: (5.700777 N, 0.033269 W) comprised a single district (Ashaiman) with a relative risk of 21.85 (Fig. 3 A). The mumps hotspots were scattered all over the country, with three of the five most likely spatial clusters occurring in the southern parts of the country (clusters 1, 2 and 5). The middle belt and the northern parts of the country had a cluster each (clusters 4 and 3, respectively).

*Space-time analysis*: The three most likely mumps space-time clusters comprised two hotspots and a cold spot. The first and third clusters which were hotspots (relative risk = 52.46 and 4.18 respectively) co-locates with their spatial counterparts (relative risk = 21.85 and 2.86 respectively), and even though none of the spatial mumps clusters were cold spots, the second most likely space-timed cluster was a cold spot (relative risk = 0.36). This large cold spot comprised 97 districts (coordinates / radius: (6.669270 N, 1.384939 W) / 163.69 km) and occurred between 2010 and 2011.

### Otitis media

*Spatial analysis*: A total of 81,156 otitis media cases were recorded in the study area during the five-year study period. The five most likely otitis media clusters comprised three hotspots and two cold spots (Fig. 4 A). The most likely cluster was a hotspot comprising a single district (Takwa Nsuaem Municipality; coordinates: (5.193130 N, 1.971290 W)) with a relative risk of 12.48. The two other hotspots (clusters 4 and 5) occurred in the northmost parts of the country with relative risks of 4.70 and 2.10, respectively (Table 1; Fig. 4 A). One of the cold spots was a large cluster (coordinates/radius: (8.385741 N, 0.066774 W) / 192.65 km) spanning the middle belt and northern parts of the country with a relative risk of 0.36. The other cold spot (coordinate/radius 5.640235 N, 1.010241 W) / 89.36 km) was found in the southern part of the country with a relative risk of 0.4.

*Space-time analysis*: The only space-time cluster detected was a hotspot covering more than half of the country (coordinates / radius: (10.605260 N, 2.593035 W) / 495.77 km) with a relative risk of 12.38. It occurred in 2014.

### Pneumonia

*Spatial analysis*: Pneumonia emerged as the VPD with the highest incidence rate (722.8 per 100,000) in the study area and for the five-year period. The five most likely pneumonia clusters in the study area shown are in Fig. 5B. Three of the five most likely clusters (clusters 1, 4 and 5) were hotspots, whiles two (clusters 2 and 3) were cold spots. The most likely cluster (coordinates/radius: (9.406780 N, 0.800510 W) / 167.94 km) was a hotspot (relative risk = 3.1) which covered extensive areas in the northern parts of the country. Almost contiguous with the most likely cluster was another hotspot (cluster 5) which spanned several districts in the middle belt of the country (coordinates/radius: (10.173846 N, 0.231248 E) / 463.30 km) and had a relative risk of 1.59. The fourth most likely cluster, also a hotspot (relative risk = 3.4), occurred in the south-western part of the country (coordinates/radius: (5.101192 N, 2.212673 W) / 28.60 km) with a relative risk of 3.4. Interestingly, the second most likely cluster, which was a cold spot (relative risk = 0.32), occurred in the middle belt (coordinates/radius: (8.703810 N, 0.054154 E) / 216.64 km), sandwiched between the two hotspots (cluster 1 and 5). The other cold spot occurred in the southern parts of Ghana (coordinates/radius: (5.465317 N, 0.617052 W) / 47.08 km) with a relative risk of 0.37.

*Space-time analysis*: The three most likely space-time pneumonia clusters comprised two cold spots and a hotspot. The only space-time pneumonia hotspot (24.83) occurred between 2010 and 2011 at a location that overlapped with spatial cluster 1 at its southern boundaries (coordinates: 8.099945 N, 1.045458 W). The second most likely space-time pneumonia cluster, which was a cold spot (relative risk = 0.24), occurred between 2012 and 2013. It co-locates with spatial cluster 3 which is also a cold spot. The last space-time cluster, also a cold spot (relative risk = 0.19), occurred between 2010 and 2011 in the middle belt of the country (coordinates/radius: (7.944419 N, 1.760507 W) / 40.31 km).

### Tetanus

*Spatial analysis*: The incidence of tetanus in Ghana for the five-year period was 2.2 cases per 100,000 between 2010 and 2014. Top five most likely clusters were all hotspots scattered across the country (Fig. 6B). The most likely cluster (coordinates: (5.721938 N, 2.346117 W) was a single district cluster (Wassa Amenfi West) in the south-western part of the country with 814 cases and a relative risk of 65.04. The fifth most likely cluster (coordinates/radius: (10.685248 N, 0.712475 W) / 19.97 km) which comprised two districts (Talensi Nabdam and Bolgatanga) was in the northern parts of the country and had a relative risk of 11.52.

*Space-time analysis*: space-time analysis revealed only two clusters, both of which were hotspots. The space-time most likely cluster occurred in 2014 (coordinates: (5.721938 N, 2.346117 W) with 814 cases and a relative risk of 327.09. The second tetanus space-time cluster occurred in 2014 (coordinates/radius: (7.705438 N, 1.553793 W) / 103.96 km) with 664 cases and a relative risk of 8.98.

### VPDs and ecological zones in Ghana

The potential effects of climate and ecology on the distribution of diseases are well known [40–43]. Thus, to gain insight into the relationship (s) between the VPDs studied and ecological zones in Ghana, this study compared the incidence of the various VPDs in different ecological zones using the Kruskal-Wallis test (Table 2). Incidence of most VPDs studied did not differ across ecological zones except for meningitis (χ^2^ (5) = 38.5521, *p* < 0.0001), otitis media (χ^2^ (5) = 15.529, *p* = 0.008) and pneumonia (χ^2^(5) = 13.163, *p* < 0.022). Meningitis was ranked highest in the Sudan Savannah (mean rank = 140.17) and lowest in the Tropical Rain Forest (mean rank = 51.50) ecological zone. Post hoc comparisons with Bonferroni correction for multiple testing (Table 2) revealed a higher incidence of meningitis in the Sudan Savannah ecological zone relative to: Tropical Rain Forest (U = 88.67, *p =* 0.001); Semi Deciduous Forest (U = -67.78, *p <* 0.0001); Transitional Zone (U = 14.22, *p* < 0.0001); Coastal Savannah (U = -60.62, *p* < 0.0001) and Guinea Savannah (U = -40.39, *p* = 0.033). The post hoc comparisons further revealed a higher incidence of meningitis in Guinea Savannah (mean rank = 99.78) relative to Semi Deciduous Forest (mean rank 72.39) ecological zones (U = 27.39, *p* < 0.023). Like meningitis, the incidence of otitis media and pneumonia were similarly higher in the Sudan Savannah ecological zone relative to Semi Deciduous Forest (Table 2).

**Table 2:** An analysis of ranks comparison of VPD incidence across ecological zones in Ghana

VPD		Mean Rank		P value
		**Urban**	**Rural**	
**Measles**		83.34	86.12	0.752
**Meningitis**		89.54	84.34	0.157
**Mumps**		71.37	89.57	0.045*
**Otitis media**		89.34	84.39	0.585
**Pneumonia**		80.39	86.97	0.468
**Tetanus**		96.66	82.29	0.102

This table shows the results of a Kruskal-Wallis H test of VPD incidence in various ecological zones. This test assumes the null hypothesis that there is no difference in the incidences among ecological zones. A significant p-value (p < 0.05) is followed by a post hoc pairwise comparison adjusted for multiple test (***italics***). *significant p values (p < 0.05); **significant p values (p < 0.0001).^#^Ecological Zones: Sudan Savannah (SS); Guinea Savannah (GS); Transition Zone (TZ); Semi Deciduous Forest (SDF); Tropical Rain Forest (TRF); Coastal Savannah (CS)

### Associations of VPD with settlement types, mining activities, and coastal proximity

#### Settlement type: the rural-urban divide

Rural-urban inequalities often have implications for infectious disease epidemiology and thus, this study compared the incidence of VPDs across rural and urban districts (Table 3). Except for mumps, which was found to be higher in predominantly rural districts (U = 1971.00 *p* = 0.045), none of the VPDs varied across the rural-urban divide (Table 3).

**Table 3:** An analysis of ranks comparison of VPD incidence among districts classified as either predominantly urban or predominantly rural

VPD		Most Likely Cluster	Year	Cluster type	Rel Risk		No of cases	Exp Cases
				*Purely Spatial*				
**Measles**	Top 5	Cluster 1	-	Hotspot	43.19		116	2.97
		Cluster 2	-	Hotspot	15.58		82	15.58
		Cluster 3	-	Hotspot	29.93		59	2.09
		Cluster 4	-	Hotspot	6.20		111	19.89
		Cluster 5	-	Cold spot	0.33		98	237.93
				*Space-time*				
	Top 3	Cluster 1	2014	Hotspot	214.2		115	0.59
		Cluster 2	2012	Hotspot	10.45		203	22.97
		Cluster 3	2010–2011	Cold spot	0.020		6	237.64
				*Purely Spatial*				
**Pneumonia**	Top 5	Cluster 1	-	Hotspot	43.19		116	2.97
		Cluster 2	-	Hotspot	15.58		82	15.58
		Cluster 3	-	Hotspot	29.93		59	2.09
		Cluster 4	-	Hotspot	6.20		111	19.89
		Cluster 5	-	Cold spot	0.33		98	237.93
				*Space-time*				
	Top 3	Cluster 1	2014	Hotspot	214.2		115	0.59
		Cluster 2	2012	Hotspot	10.45		203	22.97
		Cluster 3	2010–2011	Cold spot	0.020		6	237.64
				*Purely Spatial*				
**Meningitis**	Top 5	Cluster 1	-	Hotspot	9.17		121	12.29
		Cluster 2	-	Hotspot	12.73		82	15.58
		Cluster 3	-	Hotspot	16.80		89	6.84
		Cluster 4	-	Hotspot	13.37		82	8.21
		Cluster 5	-	Hotspot	23.18		17	0.80
				*Space-time*				
	Top 3	Cluster 1	2012	Hotspot	33.44		240	9.51
		Cluster 2	2010–2011	Cold spot	0		0	189.99
		Cluster 3	-	-			-	-
				*Purely Spatial*				
**Mumps**	Top 5	Cluster 1	-	Hotspot	21.85		7404	393.62
		Cluster 2	-	Hotspot	3.07		6955	2263.46
		Cluster 3	-	Hotspot	2.68		5743	2378.84
		Cluster 4	-	Hotspot	3.45		3442	1084.38
		Cluster 5	-	Hotspot	4.10		1788	458.31
				*Space-time*				
	Top 3	Cluster 1	2013–2014	Hotspot	52.46		7121	157.36
		Cluster 2	2010–2011	Cold spot	0.36		4246	10158.88
		Cluster 3	2014	Hotspot	4.18		2331	577.97
				*Purely Spatial*				
**Otitis**	Top 5	Cluster 1	-	Hotspot	12.48		3564	297.67
		Cluster 2	-	Cold spot	0.40		10,369	21656.47
		Cluster 3	-	Cold spot	0.36		9942	21950.18
		Cluster 4	-	Hotspot	4.70		2850	631.52
		Cluster 5	-	Hotspot	2.10		8441	4374.11
				*Space-time*				
	Top 3	Cluster 1	2014	Hotspot	12.38		46,944	8098.35
		Cluster 2	-	-	-		-	-
		Cluster 3	-	-	-		-	-
				*Purely Spatial*				
**Tetanus**	Top 5	Cluster 1	-	Hotspot	65.04		814	17.75
		Cluster 2	-	Hotspot	85.99		457	6.97
		Cluster 3	-	Hotspot	18.82		98	5.56
		Cluster 4	-	Hotspot	3.44		362	130.01
		Cluster 5	-	Hotspot	11.52		114	11.06
				*Space-time*				
	Top 3	Cluster 1	2013–2014	Hotspot	52.46		7121	157.36
		Cluster 2	2010–2011	Cold spot	0.36		4246	10158.88
		Cluster 3	-	-	-		-	-

This table shows the results of a Mann-Whitney U test of VPD incidence in districts classified as either predominantly urban or predominantly rural. This test assumes the null hypothesis that there is no difference in the incidences rates across the two settlement types. A significant p value (p < 0.05) indicates a rejection of the null hypothesis and confirms that there are differences in incidences between settlement types. Values in the settlement type columns represent mean ranks. *significant p values (p < 0.05)

#### Mining activities and incidence of VPD

To find out if districts with mining activities had elevated incidence of VPDs, districts were categorised into mining and non-mining using data from the Ghana Geological Survey Department and Toxic Sites Identification Program (TSIP). A Mann-Whitney U test found no significant difference in the incidence of VPD in mining and non-mining towns except with meningitis, which was found to be higher in non-mining areas (U = 1541.50, *p = 0.019*) (Table 4).

**Table 4:** An analysis of ranks comparison of VPD incidence among districts classified as either mining or non-mining

VPD		Ecological Zones						***P*** value
		**SS**	**GS**	**TZ**	**SDF**	**TRF**	**CS**	
		Mean Rank [Median (IQR)]						
**Meningitis**		140.17 [0.07 (0.02–0.24)]	99.78 [0.01 (0-0.02)]	75.94 [0 (0-0.01)]	72.39 [0 (0–0)]	51.50 [0 (0–0)]	79.55 [0 (0-0.03)]	< 0.0001**
		*Bonferroni adjusted p values for post hoc comparisons*:						
		SS/TRF (< 0.0001)**	SS/SDF (0.001)*	SS/CS (0.001)*	SS/TZ (< 0.0001)**	GS/SS (< 0.0001)**	SDF/GS (0.023)*	
**Pneumonia**		121.33 [ 39.7 (33.8–107)]	95.15 [41.63 (8.25–50.16)]	82.94 [29.96 (16–52)]	76.71 [26 (9.13-47)]	87.40 [31.06 (23.5-36.89)]	73.14 [31.86 (13-53.76)]	0.022*
		*Bonferroni adjusted p values for post hoc comparisons*:						
		SS/TRF (0.022)*	*SS/SDF (1.00)*	SS/CS (0.022)*	*SS/TZ (0.164)*	*GS/SS (0.385)*	*SDF/GS (1.00)*	
**Measles**		84.79 [0.02 (0-0.09)]	71.7 [0 (0-0.06)]	79.83 [0.03 (0-0.1)]	84.40 [0.01(0-0.04)]	70 [0 (0-0.01)]	92.55 [0 (0-0.06)]	0.248
**Mumps**		113.13 [2.49 (1.32-4)]	76.80 [0.71 (0.69–3.09)]	95.89 [1.68 (0.67–2.18)]	80.33 [0.98 (0.48–2.14)]	103.6 [1.64 (1.21–1.79)]	86.32 [0.79 (0.49–2.49)]	0.103
**Otitis media**		129.33 [5.02 (3.64–11.57)]	79.88 [1.73 (0.7–4.3)]	95.44 [3.7 (1.59–6.24)]	81.66 [2.5 (0.92–4.24)]	80.60 [1.78 (0.97–4.58)]	71.05 [1.73 (0.67–5.39)]	0.008*
		*Bonferroni adjusted p values for post hoc comparisons*:						
		SS/TRF (0.008)*	*SS/SDF (0.552)*	SS/CS (0.01)*	*SS/TZ (0.06)*	GS/SS (0.489)*	SDF/GS (0.014)*	
**Tetanus**		80.27 [0.01 (0-0.05)]	81.15 [0.01 (0-0.055)]	91.11 [0.015 (0-0.07)]	80.07 [0.01 (0-0.05)]	95.11 [0 (0-0.03)]	106.11 [0.015 (0-0.05)]	0.273

This table shows the results of a Mann-Whitney U test of VPD incidence in districts classified as either mining or non-mining. This test assumes the null hypothesis that there is no difference in the incidences rates across the two settlement types. A significant p value (p < 0.05) indicates a rejection of the null hypothesis and confirms that there are differences in incidences between settlement types. Values in the settlement type columns represent mean ranks

### Spatial location of the most likely clusters for all six VPDs

**Fig. 7:**
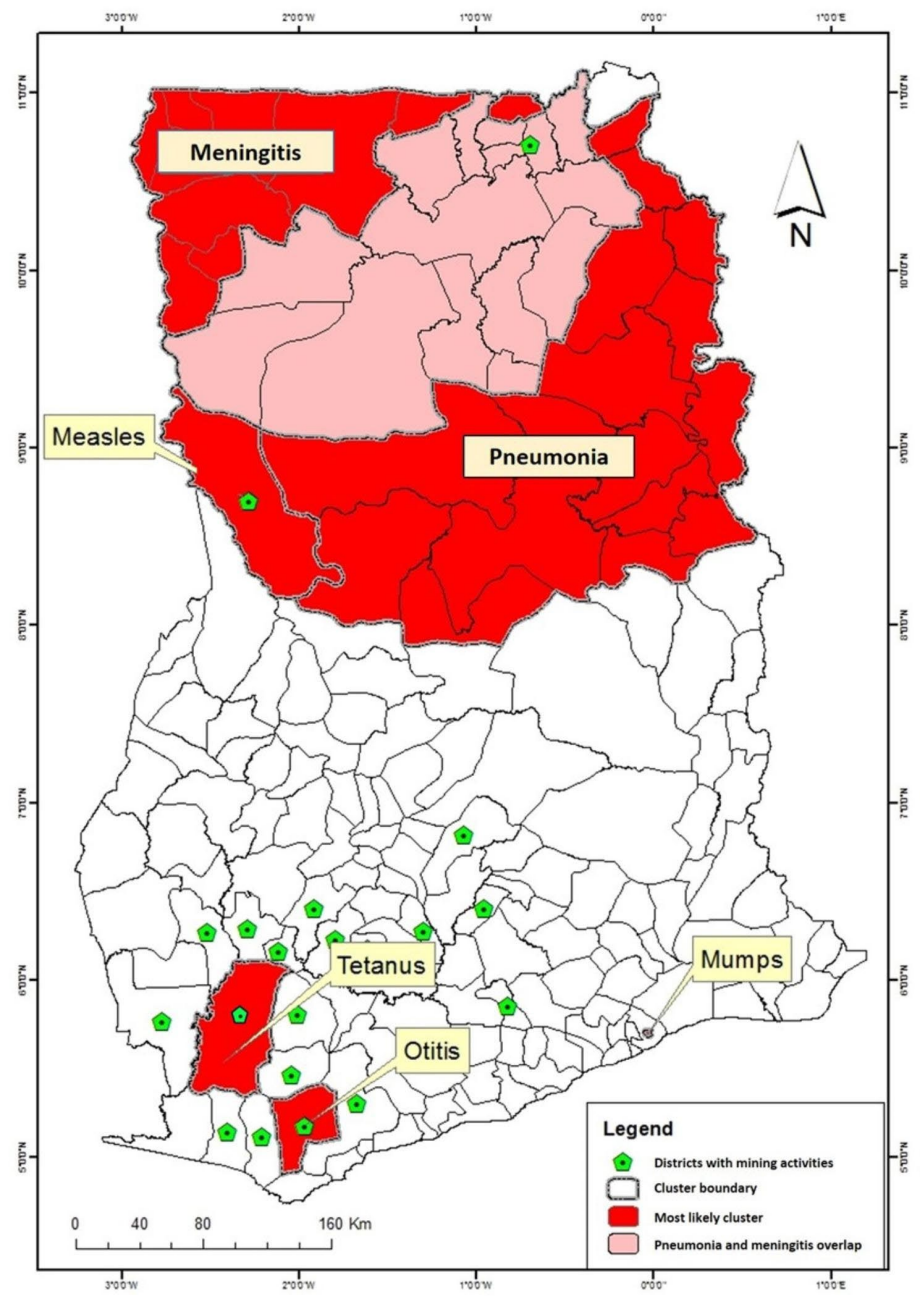
**Most likely cluster of all six VPDs. ** Most likely cluster of the six VPDs included in this study. Areas coloured red are the most likely clusters and the area coloured pink is the where meningitis and pneumonia overlap. The green diamonds indicate districts with mining activities

The most likely cluster of all six VPDs was mapped on a single choropleth to enable visualization of the relative location of each VPD cluster (Fig. 7). This enabled us to assess the burden of VPDs in the country, including overlaps. Almost every district in the northern parts of the country belonged to at least one of three high-rate clusters (meningitis, pneumonia, and measles). In addition, 15 districts in the northern parts belonged to two high-rate clusters (meningitis and pneumonia).

## Discussion

Data from this study provides insight into the clustering of six VPDs in Ghana. Spatial and space-time analysis revealed different clustering patterns for the various VPDs studied. This observation may have implications for WHO’s push for integrating VPD surveillance into a unified framework that takes advantage of existing systems to optimize outcomes [45]. The different clustering patterns of VPDs and their variegated relationships with ecology, urbanity, and land-use diversity underscore a mutual exclusivity in VPD epidemiology that may undermine this unified framework ideal. Empirical relationships observed in this study are discussed in the light of the broader public health context.

A multiplicity of factors, including complex ecological relationships, influence patterns of infectious disease transmission, and thus, the locations and ecological context of VPD clusters (hotspots and cold spots) identified in this study were deemed noteworthy. First, the preponderance of pneumococcal and meningococcal (meningitis, pneumonia, and otitis media) hotspots in the northern parts and the middle belt of Ghana was quite striking. For instance, three of the five meningitis hotspots detected covered most of the country’s Northern, Upper East and Upper West Region. Cumulatively, these three hotspots accounted for 58.63% of the meningitis cases reported in the entire country. The northernmost parts of the country lie in the African meningitis belt, which stretches from Senegal in the West to Ethiopia in the East. The country also shares its northern boundary with Burkina Faso, which is a meningitis hyperendemic country. Thus, it was unsurprising that eight districts in the meningitis hotspot shared boundaries with Burkina Faso. The only meningitis hotspots in southern Ghana occurred in the densely populated districts of Tema and Sekondi Takoradi. Although climate is a key factor driving meningitis in the meningitis belt, other factors like overcrowded housing also facilitated transmission, prolonged close contact and social interactions (mass gatherings, pilgrimages, tribe migrations, and meetings) ^21^. Thus, finding meningitis hotspots in densely populated coastal districts in the southernmost parts of Ghana is not entirely unexpected. It is noteworthy that some of the recent meningitis outbreaks in Ghana have occurred in boarding school settings [46] which is usually overcrowded and have people living in prolonged close contact with a myriad of social interactions.

The paucity of evidence supporting the impact of immunization on the incidence of VPDs further reinforces the notion that ecological and other environmental factors plausibly confound the effect of immunisation and influence VPD distribution patterns in Ghana. Although the database used in this study did not distinguish meningitis by etiologic agent, bacterial meningitis in this region is predominantly meningococcal caused by *Neisseria meningitidis* [47] or pneumococcal caused by *Streptococcus pneumoniae* [48]. Thus, authorities reckoned that the onboarding of Meningococcal A Conjugate vaccine (MenAfriVac) and pneumococcal conjugate vaccines 13 (PCV-13) onto Ghana’s EPI in November 2016 and May 2012 respectively [49–51] would reduce meningococcal and pneumococcal VPDs such as meningitis, otitis media and pneumonia. However, studies on the impact of introducing MenAfriVac and PCV-13 on disease burden in Ghana have showed no such benefit [52, 53].

Taken together, the overrepresentation of hotspots of pneumococcal and meningococcal VPDs in the northern parts of Ghana seems consistent with their association with hot climates [15, 54, 55]. This is somewhat corroborated by finding relatively higher incidence of meningitis, pneumonia and otitis media in ecological zones characterised by hot climates (i.e., Sudan Savannah and Guinea Savannah). Noteworthily, incidence of meningitis decreased as one moves from the northmost ecological zones (Sudan Savannah) to the south most ecological zones (Tropical Rain Forest and Coastal Savannah). And apparently, the same dry and windy weather that heralds meningitis outbreaks also characterises the northmost ecological zones.

The observation of a higher incidence of mumps in predominantly urban district is interpreted with care. Although health outcomes are thought to be better in urban areas, cities can also be the breeding grounds and gateway for infectious diseases [56, 57]. This makes the relationship between infectious diseases and urbanization nebulous. Studies trying to find out whether pneumococcal and meningococcal VPDs are more prevalent in urban or rural areas have so far been equivocal [58, 59]. Our cautious disposition is also borne out of our recognition of the methodological challenges faced by studies investigating the relationship between health indices and urbanity. For instance, there is still no consensus among experts on defining what is rural, urban, and peri-urban [57, 60]. Some studies have resorted to using official administrative definition of rural and urban areas, but this approach is criticised for its bluntness. Using human population density as a surrogate of urbanity also ignores the fact that a high population density with commensurate infrastructure does not necessarily represent a risk factor[60]. Plausibly, the limited definition of urbanity in this study may have obscured the complex interplay between sanitation and water supply infrastructure and their combined effect (s) on the spread and distribution of infectious diseases.

Cognisant of the potential role of mining as a human-induced land-use change that can drive infectious disease transmission dynamics [17, 18], this study hypothesized that inhabitants of ASGM communities are VPD-vulnerable due to long-term exposure to high levels of the immunotoxins used in ASGM. Although a Mann-Whitney U test did not support this hypothesis, spatial analysis revealed some telling findings. For instance, VPD hotspots in the southern parts of the country all occurred in mining districts (Tarkwa Nsuaem Municipality and Wassa Amenfi West) (Fig. 7). Mining communities may have some trappings of a transmission-enhancing environment (i.e., overcrowded housing, intense social interaction, migration etc.), but the possibility that AGSM activities in this community might have led to immunosuppression and increased VPD-vulnerability cannot be discounted [25–27, 61, 62]. Studies assessing the immune status of populations in mining areas may offer some valuable insights. Mining activities and their health consequences are often focal and thus require data with high spatial resolution, usually at the neighborhood or household level, to observe differences. However, the vastness and heterogeneity of the unit of analysis (administrative districts) may have masked the actual clustering of VPDs within districts. The relationship between VPD clustering and mining activities in the study area remains blurred and requires further studies with better spatial resolution. Perhaps VPD hotspots and cold spots that comprised single district and situations where hotspots and cold spots were contiguous need further scrutiny. The focal nature of the former and the contrasting nature of the latter make these situations uniquely suited for targeted in-depth investigations.

The findings of this study are discussed in the context of study limitations, some of which are worth highlighting. First, the disease count and population estimates used in this study could have some data quality issues, such as is common with national health datasets, particularly in LMICs [63]. This possibility notwithstanding, studies attesting to the quality, completeness and reliability of routine health information data collected in Ghana are reassuring [34, 64]. Another limitation worth highlighting is the apparent datedness of data used in this study. This study focused on a five-year period (2010–2014) to ensure data completeness – a tradeoff that invariably affects the timeliness of our findings. Under-reporting of VPDs and the spatial scale (district level) of analysis also affect the accuracy and spatial granularity of our findings. Finally, although SaTScan is a great spatial statistic tool, it is not without limitations. For instance, SaTScan’s output options are limited to text files and databases files. This limits further exploration of clusters in SaTScan itself and forces users to resort to other softwares for graphing and mapping needs[65].

## Conclusion

This study provides valuable insights into the spatial clustering of some VPDs in Ghana. It clearly shows a preponderance for VPD hotspots to occur in the northmost parts of the country and areas with semi-arid ecoclimates, but fails to establish a convincing relationship between ASGM activities and VPD transmission. The spatiotemporal signals seen in this study are difficult to interpret in the absence of rich contextual data. Besides providing public health practitioners with information on the distribution of VPD, this study provides baseline data for researchers to further explore the subject. Sequels to this study must endeavour to improve spatial resolution and focus on a better understanding of the reasons behind the spatial clustering patterns of VPDs observed in this study.

## Data Availability

Disease count data used for this study can be obtained from the Centre for Health Information Management, Ghana Health Services upon request. Population figures for each district were extracted from Ghana’s 2010 PHC data which is freely available from at https://statsghana.gov.gh/gssmain/fileUpload/pressrelease/2010_PHC_National_Analytical_Report.pdf.

## List of abbreviations

ASGM: Artisanal and Small-scale Gold Mining.
EPI: Expanded Program on Immunization.
GIS: Geographic Information Systems.
GHS: Ghana Health Service.
GHIs: Global Health Initiatives.
HICs: high-income countries.
LMICs: low and middle-income countries.
NTDs: Neglected Tropical Diseases.
PHC: Population and Housing Census.
VPDs: Vaccine-preventable diseases.

## References

[1] Rappuoli R, Pizza M, Del Giudice G, De Gregorio E (2014). Vaccines, new opportunities for a new society. Proc Natl Acad Sci U S A.

[2] Global Vaccine Action Plan Monitoring. Evaluation & Accountability: Secretariat Annual Report 2020. https://www.who.int/publications-detail-redirect/global-vaccine-action-plan-monitoring-evaluation-accountability-secretariat-annual-report-2020. Accessed 29 Jun 2021.

[3] Frenkel LD. The global burden of vaccine preventable infectious diseases in children less than 5 years of age: Can we do better? Adv Pediatr Res. 2020;:7. 10.2500/aap.2021.42.210065 PMC8677503 34474707

[4] Maurice JM, Davey S (2009). State of the world’s vaccines and immunization.

[5] Wicker S, Maltezou HC (2014). Vaccine-preventable diseases in Europe: where do we stand?. Expert Rev Vaccines.

[6] Glanz JM, McClure DL, Magid DJ, Daley MF, France EK, Hambidge SJ (2010). Parental refusal of varicella vaccination and the associated risk of varicella infection in children. Arch Pediatr Adolesc Med.

[7] Glanz JM, McClure DL, Magid DJ, Daley MF, France EK, Salmon DA (2009). Parental refusal of pertussis vaccination is associated with an increased risk of pertussis infection in children. Pediatrics.

[8] Phadke VK, Bednarczyk RA, Salmon DA, Omer SB (2016). Association Between Vaccine Refusal and Vaccine-Preventable Diseases in the United States. JAMA.

[9] Guignard A, Praet N, Jusot V, Bakker M, Baril L (2019). Introducing new vaccines in low- and middle-income countries: challenges and approaches. Expert Rev Vaccines.

[10] Pang T. Vaccination in Developing Countries: Problems, Challenges and Opportunities.:7.

[11] Kambarami R, Nathoo K, Nkrumah F, Pirie D (1991). Measles epidemic in Harare, Zimbabwe, despite high measles immunization coverage rates. Bull World Health Organ.

[12] Qin W, Wang Y, Yang T, Xu X-K, Meng X-M, Zhao C-J (2019). Outbreak of mumps in a student population with high vaccination coverage in China: time for two-dose vaccination. Hum Vaccines Immunother.

[13] Sala-Farré M-R, Arias-Varela C, Recasens-Recasens A, Simó-Sanahuja M, Muñoz-Almagro C, Pérez-Jové J (2015). Pertussis epidemic despite high levels of vaccination coverage with acellular pertussis vaccine. Enferm Infecc Microbiol Clin.

[14] Mahmud AS, Martinez PP, He J, Baker RE (2020). The Impact of Climate Change on Vaccine-Preventable Diseases: Insights From Current Research and New Directions. Curr Environ Health Rep.

[15] Sultan B, Labadi K, Guégan J-F, Janicot S (2005). Climate Drives the Meningitis Epidemics Onset in West Africa. PLOS Med.

[16] Codjoe SNA, Nabie VA (2014). Climate Change and Cerebrospinal Meningitis in the Ghanaian Meningitis Belt. Int J Environ Res Public Health.

[17] Patz JA, Daszak P, Tabor GM, Aguirre AA, Pearl M, Epstein J (2004). Unhealthy Landscapes: Policy Recommendations on Land Use Change and Infectious Disease Emergence. Environ Health Perspect.

[18] Patz JA, Olson SH, Uejio CK, Gibbs HK. Disease emergence from global climate and land use change. Med Clin North Am. 2008;92:1473–91, xii. 10.1016/j.mcna.2008.07.007 19061763

[19] Global strategy for comprehensive Vaccine-Preventable Disease (VPD) surveillance. https://www.who.int/publications/m/item/global-strategy-for-comprehensive-vaccine-preventable-disease-(vpd)-surveillance. Accessed 30 Jun 2021.

[20] Threats I of M (US) F on M. Climate, Ecology, and Infectious Disease. National Academies Press (US); 2008.

[21] Hughes RM, Omernik JM (1999). Ecological regions (ecoregions). Environmental Geology.

[22] Plans-Rubió P. Vaccination Coverage for Routine Vaccines and Herd Immunity Levels against Measles and Pertussis in the World in 2019. Vaccines. 2021;9. 10.3390/vaccines9030256 PMC7999208 33805681

[23] Basu N, Clarke E, Green A, Calys-Tagoe B, Chan L, Dzodzomenyo M (2015). Integrated Assessment of Artisanal and Small-Scale Gold Mining in Ghana—Part 1: Human Health Review. Int J Environ Res Public Health.

[24] UNEP U. Global Mercury Assessment 2013: Sources, Emissions, Releases and Environmental Transport. UNEP Chem Branch Geneva Switz. 2013.

[25] Dangleben NL, Skibola CF, Smith MT (2013). Arsenic immunotoxicity: a review. Environ Health.

[26] Ahmed S, Moore SE, Kippler M, Gardner R, Hawlader MDH, Wagatsuma Y (2014). Arsenic exposure and cell-mediated immunity in pre-school children in rural bangladesh. Toxicol Sci Off J Soc Toxicol.

[27] Biswas R, Ghosh P, Banerjee N, Das JK, Sau T, Banerjee A (2008). Analysis of T-cell proliferation and cytokine secretion in the individuals exposed to arsenic. Hum Exp Toxicol.

[28] Pollard KM, Hultman P (1997). Effects of mercury on the immune system. Met Ions Biol Syst.

[29] Fobil JN, Levers C, Lakes T, Loag W, Kraemer A, May J (2012). Mapping urban malaria and diarrhea mortality in Accra, Ghana: evidence of vulnerabilities and implications for urban health policy. J Urban Health Bull N Y Acad Med.

[30] Osei FB, Duker AA (2008). Spatial and demographic patterns of Cholera in Ashanti region - Ghana. Int J Health Geogr.

[31] Nilima null, Kamath A, Shetty K, Unnikrishnan B, Kaushik S, Rai SN (2018). Prevalence, patterns, and predictors of diarrhea: a spatial-temporal comprehensive evaluation in India. BMC Public Health.

[32] Kreuels B, Kobbe R, Adjei S, Kreuzberg C, von Reden C, Bäter K (2008). Spatial Variation of Malaria Incidence in Young Children from a Geographically Homogeneous Area with High Endemicity. J Infect Dis.

[33] Rodríguez-Morales AJ, Orrego-Acevedo CA, Zambrano-Muñoz Y, García-Folleco FJ, Herrera-Giraldo AC, Lozada-Riascos CO (2015). Mapping malaria in municipalities of the Coffee Triangle region of Colombia using Geographic Information Systems (GIS). J Infect Public Health.

[34] Awine T, Malm K, Peprah NY, Silal SP (2018). Spatio-temporal heterogeneity of malaria morbidity in Ghana: Analysis of routine health facility data. PLoS ONE.

[35] Kiani B, Raouf Rahmati A, Bergquist R, Hashtarkhani S, Firouraghi N, Bagheri N (2021). Spatio-temporal epidemiology of the tuberculosis incidence rate in Iran 2008 to 2018. BMC Public Health.

[36] Clarke KC, Osleeb JP, Sherry JM, Meert JP, Larsson RW (1991). The use of remote sensing and geographic information systems in UNICEF’s dracunculiasis (Guinea worm) eradication effort. Prev Vet Med.

[37] Braddock M, Lapidus G, Cromley E, Cromley R, Burke G, Banco L (1994). Using a geographic information system to understand child pedestrian injury. Am J Public Health.

[38] Shabanikiya H, Hashtarkhani S, Bergquist R, Bagheri N, VafaeiNejad R, Amiri-Gholanlou M (2020). Multiple-scale spatial analysis of paediatric, pedestrian road traffic injuries in a major city in North-Eastern Iran 2015–2019. BMC Public Health.

[39] Kulldorff M (1997). A spatial scan statistic. Commun Stat - Theory Methods.

[40] Ghana Statistical Service. 2010 Population and Housing Census: Summary Report and Final Results. 2012.

[41] Ahanhanzo CD, Huang XX, Le Gargasson J-B, Sossou J, Nyonator F, Colombini A, et al. Determinants of routine immunization costing in Benin and Ghana in 2011. Vaccine. 2015;33, Supplement 1:A66–71. 10.1016/j.vaccine.2014.12.069 25919178

[42] Vandenbroucke JP, Pearce N (2012). Incidence rates in dynamic populations. Int J Epidemiol.

[43] Kulldorff M, Nagarwalla N (1995). Spatial disease clusters: Detection and inference. Stat Med.

[44] Reed College | Stata Help | The Theory Behind Mann-Whitney U tests & Kruskal-Wallis ANOVAs. 2015. http://academic.reed.edu/psychology/stata/analyses/nonparametric/kruskal-wallacetheory.html. Accessed 7 Oct 2015.

[45] Hyde TB, Andrus JK, Dietz VJ, Andrus JK, Hyde TB, Lee CE (2013). Critical issues in implementing a national integrated all-vaccine preventable disease surveillance system. Vaccine.

[46] Kwarteng A, Amuasi J, Annan A, Ahuno S, Opare D, Nagel M (2017). Current meningitis outbreak in Ghana: Historical perspectives and the importance of diagnostics. Acta Trop.

[47] Greenwood B (1999). Manson Lecture: Meningococcal meningitis in Africa. Trans R Soc Trop Med Hyg.

[48] O’Brien KL, Wolfson LJ, Watt JP, Henkle E, Deloria-Knoll M, McCall N (2009). Burden of disease caused by Streptococcus pneumoniae in children younger than 5 years: global estimates. The Lancet.

[49] International Vaccine Access Center (IVAC). Johns Hopkins Bloomberg School of Public Health) VIEW-hub. http://www.view-hub.org/viz/. Accessed 6 Apr 2020.

[50] Dayie NT, Arhin RE, Newman MJ, Dalsgaard A, Bisgaard M, Frimodt-Møller N (2013). Penicillin resistance and serotype distribution of Streptococcus pneumoniaein Ghanaian children less than six years of age. BMC Infect Dis.

[51] Bwaka A, Bita A, Lingani C, Fernandez K, Durupt A, Mwenda JM (2019). Status of the Rollout of the Meningococcal Serogroup A Conjugate Vaccine in African Meningitis Belt Countries in 2018. J Infect Dis.

[52] Dayie NTKD, Tettey EY, Newman MJ, Bannerman E, Donkor ES, Labi A-K (2019). Pneumococcal carriage among children under five in Accra, Ghana, five years after the introduction of pneumococcal conjugate vaccine. BMC Pediatr.

[53] Trotter CL, Lingani C, Fernandez K, Cooper LV, Bita A, Tevi-Benissan C (2017). Impact of MenAfriVac in nine countries of the African meningitis belt, 2010–15: an analysis of surveillance data. Lancet Infect Dis.

[54] García-Pando CP, Thomson MC, Stanton MC, Diggle PJ, Hopson T, Pandya R (2014). Meningitis and climate: from science to practice. Earth Perspect.

[55] Palmgren H. Meningococcal disease and climate. Glob Health Action. 2009;2:2061. 10.3402/gha.v2i0.2061 PMC2799239 20052424

[56] Havemann K. Report to the WHO Commission on Social Determinants of Health from the Knowledge Network on Urban Settings Hub: WHO Kobe Centre, Kobe, Japan.:70.

[57] Connolly C, Keil R, Ali SH (2021). Extended urbanisation and the spatialities of infectious disease: Demographic change, infrastructure and governance. Urban Stud.

[58] Floyd RF, Federspiel CF, Schaffner W (1974). Bacterial Meningitis in Urban and Rural Tennessee. Am J Epidemiol.

[59] Giannakopoulos I, Leotsinidis M, Diamantopoulos S, Makrakis K, Ellina A, Giannakopoulos A (2008). Rarity of bacterial and viral meningitis in areas of Western Greece with fewer than 2,000 inhabitants. Jpn J Infect Dis.

[60] World Bank. World Development Report 2009: Reshaping Economic Geography. 2009.

[61] Engström K, Wojdacz TK, Marabita F, Ewels P, Käller M, Vezzi F (2016). Transcriptomics and methylomics of CD4-positive T cells in arsenic-exposed women. Arch Toxicol.

[62] Ahmed S, Mahabbat-e Khoda S, Rekha RS, Gardner RM, Ameer SS, Moore S (2011). Arsenic-associated oxidative stress, inflammation, and immune disruption in human placenta and cord blood. Environ Health Perspect.

[63] Healthcare’s. “Big Data” Challenge. 2015. http://www.ajmc.com/journals/issue/2013/2013-1-vol19-n7/healthcares-big-data-challenge. Accessed 8 Dec 2015.

[64] Amoakoh-Coleman M, Kayode GA, Brown-Davies C, Agyepong IA, Grobbee DE, Klipstein-Grobusch K (2015). Completeness and accuracy of data transfer of routine maternal health services data in the greater Accra region. BMC Res Notes.

[65] Robertson C, Nelson TA (2010). Review of software for space-time disease surveillance. Int J Health Geogr.

